# mHealth for the Self-management of Knee Osteoarthritis: Scoping Review

**DOI:** 10.2196/38798

**Published:** 2023-05-08

**Authors:** Takashi Kitagawa, Masateru Hayashi

**Affiliations:** 1 Department of Physical Therapy School of Health Sciences Shinshu University Matsumoto Japan; 2 Department of Rehabilitation Hanamizuki Orthopedics Sports Clinic Kiyosu Japan

**Keywords:** knee osteoarthritis, mobile health, mHealth, self-management, knee joint, scoping review, pain, physical function, quality of life, mobile phone app, patient education

## Abstract

**Background:**

Educating patients on the self-management of knee osteoarthritis (OA) reportedly reduces pain, improves activities of daily living, and even reduces health care costs.

**Objective:**

This scoping review will summarize the current evidence on mobile health (mHealth) and smartphone app–based disease self-management for patients with knee OA.

**Methods:**

PubMed, Web of Science, the Cochrane Central Register of Controlled Trials, and CINAHL were systematically searched in May 2021 using the keywords “knee osteoarthritis,” “mobile health,” and “self-management.” Studies that investigated patients with knee OA based on radiography or clinical diagnosis were included. The following criteria were applied to the mobile phone apps included in the search-derived studies: the ability to (1) record and manage symptoms, (2) provide patient education, and (3) guide and record activities of daily living. Studies eligible for inclusion in this scoping review were interventional trials or observational studies published in English.

**Results:**

This scoping review included 8 reports, of which 3 were randomized controlled trials and 1 was a conference abstract. Most studies provided data on the outcomes of pain, physical function, and quality of life.

**Conclusions:**

An increasing number of reports are addressing the effectiveness of mHealth in patients with knee OA, and the data suggest that mHealth efficacy is similar to conventional management of health.

**International Registered Report Identifier (IRRID):**

RR2-10.17504/protocols.io.buuxnwxn

## Introduction

Knee osteoarthritis (OA) is one of the causes of reduced life expectancy in many countries around the world [1]. It is essential that the symptoms of knee OA be managed by patients themselves to reduce disability-adjusted life years and control rising medical costs. In recent years, the role of eHealth, mobile health (mHealth), and internet-based interventions in the treatment of knee arthritis have been receiving increasing attention [2,3]. Using these technologies, continuous patient follow-up is possible even after discharge from the hospital. A systematic review reported that digital self-management interventions for patients with knee OA significantly improved pain and physical function compared to conventional therapy [4]. mHealth supports self-management by allowing patients to record their pain levels and physical activities over time using a mobile app [5], and feedback can be sent based on patient-reported data. Apps can be personalized to motivate patients to continue exercise and other activities [6]. In fact, the use of short message services in patients with various chronic diseases has been reported to help improve self-management and treatment compliance [7]. Similar effects are expected for the self-management of knee OA, and interventional trials are increasingly being registered to investigate the impact of these technologies.

There are several advantages to mHealth over conventional interventions. While there is a limit to the number of patients and procedures that a single medical professional can manage daily, there is theoretically no limit to the number of therapeutic interventions that can be performed using apps. Furthermore, patients who have geographical barriers to accessing medical care, such as those living in mountainous or rural areas, can receive medical care at home, thus reducing the need for hospital visits and potentially reducing medical costs [8]. If mHealth is proven useful and becomes widely adopted, it will allow more patients to enjoy high-quality and consistent medical care [9]. In addition, in the setting of a global pandemic caused by a new infectious disease, contact with others can be minimized; thus, mHealth is also expected to play a role in infection control.

As described above, the widespread use of mHealth apps that assist in the self-management of knee OA could reduce the burden of medical costs on individuals, reduce social security costs, and reduce socioeconomic disparities in medical care. However, the development of mHealth apps for patients with knee OA is still in its infancy compared with mHealth apps for other diseases. In addition, some existing studies include patients with hip and knee OA as mixed participants, and this may increase data heterogeneity [10-14]. As a result, it is currently difficult to demonstrate the effectiveness of mHealth apps for knee OA. Therefore, there is a need to understand and summarize the current evidence and identify issues with existing technologies. To our knowledge, there have been no high-quality systematic reviews or scoping reviews published thus far that address the use of apps for knee OA. It is also important to summarize the definitions and mainstreaming of terms related to mHealth research for knee OA. This scoping review aims to summarize the current evidence on mHealth and app-based disease self-management for patients with knee OA.

## Methods

The protocol for this review was registered with protocols.io prior to commencement [15].

### Eligibility Criteria

Patients with unilateral or bilateral knee OA were included, with a diagnosis based on the physician’s assessment or radiography. Self-reported cases were excluded. There were no age or sex restrictions. Patients were included if their disease severity corresponded to grades I-IV of the Kellgren-Lawrence classification system.

Studies using apps with features that fit one or more of the following criteria were eligible for inclusion in this scoping review: (1) documenting or self-managing knee OA-related pain and other symptoms, (2) providing patient education, and (3) instructing or recording activities of daily living (such as exercise and diet). According to a previous study, self-management activities include maintaining good health and preventing adverse events, interacting with health care providers, improving self-monitoring, managing symptoms of knee OA, developing problem-solving skills, making decisions, using resources, forging partnerships with providers, and taking action [4]. Patient education was defined as content (videos and documents) that provided patients with knowledge on the pathogenesis of OA, treatment information, specific strategies to deal with pain, and appropriate exercise [16]. Studies on decision-making related to knee OA or assessing joint function were excluded. Additionally, studies were excluded if patients with diseases other than knee OA (such as hip OA) were included, as the results of knee OA could not be isolated from those of other diseases in such reports.

Many studies have investigated the effects of conservative management for knee OA using pain scales, functional assessments, and quality of life (QoL) measurements. In other words, the main goals for the management of knee OA should be pain relief, improvement in physical function, and enhancement of QoL [17]. Therefore, this scoping review summarizes the results of the included studies by using the 3 categories of pain, physical function, and QoL.

There were no restrictions based on region, race, or sex in the study selection. The search results were limited to papers published in peer-reviewed journals in English. Protocol papers, conference abstracts, interventional studies, and observational studies, including exploratory studies, were included. Systematic reviews or meta-analyses, case series, and case reports were excluded.

### Search Strategy

The following databases were used to conduct an electronic search: PubMed, Web of Science, the Cochrane Central Register of Controlled Trials, and CINAHL. A comprehensive search strategy for each of the 4 databases was developed using the words contained in the titles and abstracts of the relevant articles and the indexed terms from the reports (see Multimedia Appendix 1). The search period was from January 2007 (approximately the start of the smartphone era) to April 2021. The primary search was conducted in May 2021, followed by an updated electronic search and a manual search (mostly a citation search) in January 2022.

### Study Selection

**Figure 1:**
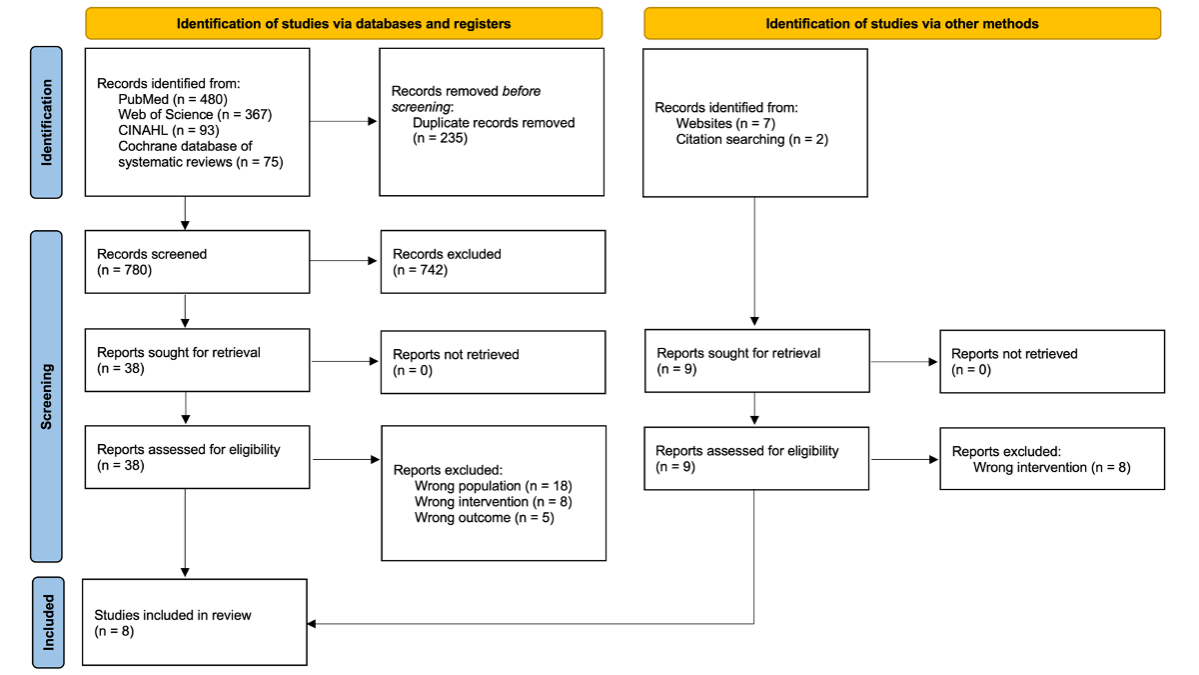
A flowchart including searches of databases, registers, and other sources.

Citations were collated and uploaded to the Qatar Computing Research Institute, Ar Rayyan, Qatar [18], and duplicates were removed. Following a pilot test, 2 independent reviewers conducted a screening based on the eligibility criteria. This process was carried out in two stages: (1) during the first screening stage, titles, and abstracts were screened for inclusion or exclusion and (2) during the second screening stage, the full text was screened and evaluated. For studies excluded in the second screening stage, the reasons for exclusion were recorded. An independent third reviewer resolved any disagreements between the 2 reviewers. A PRISMA (Preferred Reporting Items for Systematic Reviews and Meta-Analysis) [19] format flow diagram shows the search results and study inclusion process (Figure 1).

### Data Extraction

The data extraction was performed by 2 independent reviewers using a spreadsheet. The extracted data included information on the first author, year of publication, country of origin, study design, population, sample size, intervention type, comparator, outcomes, time points of follow-up assessment, key findings of relevance to this scoping review, and the conclusion. Any discrepancies between the 2 reviewers were discussed and finalized by a third reviewer.

### Data Analysis and Presentation

The outcomes identified in the literature were analyzed in 3 categories of pain, physical function, and QoL.

## Results

Our database searches identified 1015 records, and after removing duplicates, 780 titles and abstracts were screened. Of these, 742 records failed to meet our eligibility criteria. Thirty-eight full-text articles that passed the primary eligibility screening and an additional 9 studies, including those identified through a manual search, were also screened. Finally, 8 studies were selected for inclusion in this scoping review [20-27] (Figure 1).

The years of publication of the included studies were 2017 (n=1) [22], 2019 (n=1) [20], 2020 (n=3) [23,25,26], and 2021 (n=3) [21,24,27]. There were 3 randomized controlled trials (RCTs) [21,23,24], 4 RCT protocols [22,25-27], and 1 conference abstract on an RCT [20]. The studies were conducted in the United States, the Netherlands, Germany, Australia, Turkey, China, Pakistan, and Taiwan. Several studies included patients with hip OA [25,26], and 4 included patients before or after total knee replacement [22,23,25,26]. Three studies focused on patients with obesity and knee OA [21,24,25]. In terms of mHealth and the apps evaluated, 7 studies included mHealth or apps on exercise therapy such as strength training [20-24,26,27], 6 involved patient education [20-23,25,26], and 2 were related to dietary advice [21,25]. Word clouds generated by the titles and abstracts of the 8 studies are shown in Multimedia Appendix 2, and a summary of our findings is shown in Tables 1 and 2.

The outcomes presented by the included studies are shown in Table 3.

The numerical rating scale, visual analog scale, and Western Ontario and McMaster Universities Osteoarthritis Index (WOMAC) pain subscales were used as pain-related outcomes [20,23,24,26,27]. Outcomes related to physical function included the Knee injury and Osteoarthritis Outcome Score-Physical Function Shortform (KOOS-PS), the WOMAC score, and the Timed Up and Go (TUG) test [20-27]. The RAND 36-Item Short Form Health Survey (SF-36) and other similar surveys were used as outcomes related to QoL [20,22,23,26,27]. In reports of 4 RCTs examining the effectiveness of mHealth for each outcome, most RCTs, particularly those using exercise therapy interventions, showed benefits in pain and physical function outcomes. On the other hand, no significant effect of mHealth on QoL was observed in 4 trials.

**Table 1:** Studies included in this scoping review.

Study	Year	Country	Study design	Population	Sample size, N	Intervention type	Comparator
Hsu et al [21]	2021	Taiwan	RCT^a^	Obese knee OA^b^ (mild-moderate)	66	Both home-based nutritional and telemedicine-based resistance exercise	Either home-based nutritional or telemedicine-based resistance exercise
Rafiq et al [24]	2021	Pakistan	RCT	Knee OA overweight or obese (KL^c^ grade 2-3)	114	Lower limb rehabilitation protocol (mHealth^d^) and instructions of daily care	Lower limb rehabilitation protocol and instructions of daily care
Pronk et al [23]	2020	Netherlands	RCT	TKR^e^ (American Society of Anesthesiologists score I-II, BMI ≤35)	76	PainCoach (app) and usual care	Usual care
Aydogdu et al [20]	2019	Turkey	RCT (congress report)	Knee OA (KL grade 2-3), age 45-65 years	40	A mobile phone–based home exercise training program	A brochure-phone–based home exercise training program
Wang et al [27]	2021	China	RCT (protocol)	Aged ≥50 years with symptomatic knee OA	110	Neuromuscular exercise, education	Quadriceps exercise, education
Seward et al [25]	2020	United States	RCT (protocol)	TJA^f^	60	A telemedicine web-based or smartphone app (Nutrimedy) with video calls and unlimited in-app text messaging	Clinical standard of care
Stauber et al [26]	2020	Germany	RCT (protocol)	TKA^g^/THA^h^	160	Standard care and RECOVER-E (app)	Standard care
Hussain et al [22]	2017	Australia	RCT (protocol)	TKR	320	TKR Platform (app and wearable)	Usual care

^a^RCT: randomized controlled trial.

^b^OA: osteoarthritis.

^c^KL: Kellgren and Lawrence.

^d^mHealth: mobile health.

^e^TKR: total knee replacement.

^f^TJA: total joint arthroplasty.

^g^TKA: total knee arthroplasty.

^h^THA: total hip arthroplasty.

**Table 2:** Outcomes and key findings of studies included in this scoping review.

Author	Outcomes				Timepoints			Key findings		Conclusion
	Pain	Physical function	QoL^a^	Baseline		Follow-up				
Hsu et al [21]	—^b^	WOMAC^c^ and TUG^d^ test	—	✔		12 weeks	MCIDs^e^ were observed in all 3 groups on each outcome		Individual diet control intervention combined with telemedicine-based resistance exercise intervention significantly improved lower-limb functional performance.	
Rafiq et al [24]	WOMAC pain subscale	TUG test, Patient-Specific Functional Scale, and Katz Index of Independence in ADL^f^	—	✔		3 months	Rehabilitation group with mHealth^g^ had less knee pain, better functional activity, faster mobility, and better improvement in ADL scores.		The importance of mHealth was revealed in rehabilitation programs for overweight and obese patients with knee OA^h^.	
Pronk et al [23]	VAS^i^	KOOS^j^-Physical Function Short-form and OKS^k^	EQ-5D-3L	✔		Postoperatively 1-14 days, 1 month	The VAS pain score during activity significantly decreased 4.1 times faster in the active PainCoach subgroup.		Active use of the PainCoach app leads to a further improvement of pain control.	
Aydogdu et al [20]	VAS	WOMAC and Berg Balance Scale	SF-36^l^	✔		3 weeks	No significant differences were found in any of patient outcome variables between the groups.		A mobile phone–based home exercise training program is not superior to brochure-based home exercise training program in terms of patient outcomes over a 3-week period.	
Wang et al [27]	NRS^m^, WOMAC pain subscale	WOMAC physical function subscale, 6-minute walk test, TUG test, and Stanford brief activity survey	SF-36	✔		4, 8, 12, 16, 20, or 24 weeks	N/A^n^		This study may provide promising insights in terms of exercise therapy optimization for people with knee OA or other chronic pain within a psychosocial framework.	
Seward et al [25]	—	KOOS	—	✔		6, 12, and 24 weeks	N/A		This will be the first study to assess preoperative weight loss in patients with severe obesity anticipating orthopedic surgery using a remote dietitian and mobile app intervention aimed at helping patients become eligible for total joint arthroplasty.	
Stauber et al [26]	NRS, KOOS subscale (pain)	KOOS subscales (symptoms, ADL and Sport or Rec) and IPAQ^o^	KOOS subscales (QoL)	Before surgery: 0-6 weeks		1 day, 7 days, and 3 months after surgery	N/A		This is the first study to investigate the effect of an evidence-based mobile app on patient reported outcomes after joint replacement.	
Hussain et al [22]	—	OKS and ROM^p^	SF-36	4 weeks before surgery and immediately before surgery		12 weeks and 52 weeks after surgery	N/A		This trial investigated the clinical and behavioral efficacy of the app and the impact of a total knee replacement in terms of service satisfaction, acceptance, and economic benefits of the provision of digital services.	

^a^QoL: quality of life.

^b^Not available.

^c^WOMAC: Western Ontario and McMaster Universities Osteoarthritis Index.

^d^TUG: Timed Up and Go.

^e^MCID: minimal clinically important difference.

^f^ADL: activity of daily living.

^g^mHealth: mobile health.

^h^OA: osteoarthritis.

^i^VAS: visual analog scale.

^j^KOOS: Knee Injury and Osteoarthritis Outcome Score.

^k^OKS: Oxford Knee Score.

^l^SF-36: RAND 36 Item Short-Form Health Survey.

^m^NRS: numerical rating scale.

^n^N/A: not applicable.

^o^IPAQ: International Physical Activity Questionnaire.

^p^ROM: range of motion.

**Table 3:** Counts of each performance outcome studied.

Outcome types and details		Count, n
**Pain**		
	NRS^a^	2 [26,27]
	VAS^b^	2 [20,23]
	WOMAC^c^ pain subscale	2 [24,27]
	KOOS^d^ subscale (pain)	1 [26]
**Physical function**		
	TUG^e^ test	3 [21,24,27]
	OKS^f^	2 [22,23]
	WOMAC	2 [20,21]
	Berg Balance Scale	1 [20]
	International Physical Activity Questionnaire	1 [26]
	Katz Index of Independence in ADL^g^	1 [24]
	KOOS	1 [25]
	KOOS subscales (physical function)	1 [23]
	KOOS subscales (symptoms, ADL, and Sport & Rec)	1 [26]
	Patient-Specific Functional Scale	1 [24]
	ROM^h^	1 [22]
	Six-minute walk test	1 [27]
	Stanford brief activity survey	1 [27]
	WOMAC physical function subscale	1 [27]
**QoL^i^**		
	SF-36^j^	3 [20,22,27]
	KOOS subscale (QoL)	1 [26]
	The EuroQol-5 Dimensions 3-Level version questionnaire	1 [23]

^a^NRS: numerical rating scale.

^b^VAS: visual analog scale.

^c^WOMAC: Western Ontario and McMaster Universities Osteoarthritis Index.

^d^KOOS: Knee Injury and Osteoarthritis Outcome Score.

^e^TUG: Timed Up and Go.

^f^OKS: Oxford Knee Score.

^g^ADL: activity of daily living.

^h^ROM: range of motion.

^i^QoL: quality of life.

^j^SF-36: RAND 36 Item Short-Form Health Survey.

## Discussion

### Principal Findings

Articles published within the past 5 years were found to be relevant to this scoping review, suggesting that the majority of relevant literature is concentrated in recent years. Of the 8 studies included, 3 studies were RCTs [21,23,24], 1 was a conference abstract [20], and the remaining 4 were RCT protocols [22,25-27]. Using pain, physical function, and QoL as outcomes, mHealth was shown to be almost as effective as standard therapy in all RCTs. Effective mHealth interventions included exercise therapy, patient education, and dietary advice. The interventions varied in frequency, intensity, duration, and type, but most mHealth-enabled interventions improved associated outcomes effectively.

### Pain Outcomes

The effectiveness of mHealth for improving pain was examined in 5 studies, including the 3 RCTs and 1 conference abstract that examined the differences in effectiveness compared with a control group. In 1 RCT, there was no significant difference in pain scores between the 2 groups (mHealth versus conventional therapy). However, subgroup analysis in patients who actively used the mHealth app showed improvements in pain scores [23]. In the conference abstract, outcomes were compared between a home exercise training group that used mHealth and a brochure. Both groups showed significant improvement in the visual analog scale; however, there was no significant difference between the 2 groups [20]. Another RCT provided instructions on daily therapy without using mHealth in the control group. In this study, there was a greater improvement in WOMAC pain scores in the intervention group using mHealth [24]. However, the follow-up periods of the 2 included RCTs and 1 conference abstract were 1 month, 3 months, and 3 weeks, respectively; studies that examine outcomes for more extended periods are warranted.

Previous studies on mHealth with a patient, intervention, comparison, outcome (PICO) model, analogous to this review, have also reported improved pain outcomes for patients using mHealth compared with the control group [14,28,29]. Although we note that the patients and interventions differ slightly from those in our review, no significant differences in pain outcomes between mHealth and control groups have been reported [10,12]. Future systematic reviews should more precisely define their PICO models in order to deliver more objective assessments of efficacy.

### Physical Function Outcomes

All 8 studies examined the effectiveness of mHealth in improving physical function, and the 3 RCTs and 1 conference abstract examined the difference in effectiveness between the mHealth and control groups. In one RCT that compared mHealth with conventional therapy, there was no significant difference in KOOS-PS scores between the 2 groups. However, in a subgroup analysis of patients who actively used mHealth, there was a significant improvement in KOOS-PS scores [23]. In the conference abstract that compared home exercise training groups using a mobile phone and a brochure, there was a significant improvement in the Berg Balance Scale and WOMAC scores in both groups before and after the intervention; however, there was no significant difference between the 2 groups [20]. Another RCT reported significant improvements in the TUG test and the Katz Index of Independence in Activities of Daily Living in the group using mHealth [24]. The remaining RCT involved 3 treatment groups: diet, exercise, and a combination of diet and exercise. All 3 intervention strategies were associated with significant improvements in WOMAC scores and the TUG test [21]. However, the follow-up periods of the included 3 RCTs and 1 conference abstract were 1 month, 3 months, 12 weeks, and 3 weeks, respectively. Future studies should examine the long-term effectiveness of mHealth interventions in improving physical function. It should also be noted that one 3-arm RCT did not have a strict control group [21]. As such, the effectiveness of diet control and exercise therapy cannot be compared.

In another study, WOMAC scores improved after 24 weeks of mHealth intervention [28]. Another report on concomitant hyaluronate treatment showed an increase in walking speed and activity after 90 days of mHealth intervention [29]. Conversely, in a similar study on hip OA, the mHealth intervention group showed almost no improvement in physical function compared with that of the control group [10,12]. Due to the variety of outcomes associated with physical function, researchers should delineate outcomes carefully before conducting a systematic review. Through this review, we have identified KOOS-PS and WOMAC as the common measures used in the assessment of knee joint function. In studies with these outcomes, rather than simply assessing statistical significance, it is essential to consider whether there is an effect beyond the minimal clinically important difference.

### QoL Outcomes

The efficacy of mHealth in improving QoL was examined in 5 studies. One RCT and a conference abstract examined the difference in QoL between an mHealth group and a control group. The RCT compared mHealth with conventional care. There was no significant difference between the 2 groups in the results of the EQ-5D questionnaire. In a subgroup analysis of patients who actively used the app, there was also no improvement in the EQ-5D results [23]. The conference abstract compared a home exercise training group between a mobile phone and a brochure. Both groups showed significant improvements in the SF-36 questionnaire before and after the intervention; however, there was no significant difference between the 2 groups [20]. The follow-up periods for the included RCT and the conference abstract were 1 month and 3 weeks, respectively, so the effects of the intervention may have been temporary. As with the other outcomes described above, it would be appropriate to conduct future studies to examine the long-term effects of mHealth on QoL.

Although participants and interventions were not the same, other similar studies on mHealth have reported no significant differences in QoL outcomes between mHealth and control groups [12,30]. A systematic review with a specific PICO model should be used to determine the effectiveness of mHealth on QoL.

### Recommendations for Future Research

In recent years, the number of RCTs on mHealth in the management of knee OA has increased. Some protocol papers have also been published [31,32]. Although not included in this review, there are a number of other studies that have recruited participants through websites, and the presence or absence of knee OA was self-reported in the studies [12,33]. The recent data generated from the extant literature can guide the direction of future RCTs and systematic reviews.

### Limitations

There are 3 primary limitations to this study. First, the definition of mHealth as a form of medical intervention was not presented in detail. As a result, the scope of mHealth in the included studies was heterogeneous. In the future, mHealth interventions should be more rigorously defined. Second, the risk of bias and the quality of the reviews were not assessed. Although these evaluations are not essential in scoping reviews, readers should be aware of this limitation. Third, the studies did not consider the severity of knee OA in participants, and as a result, this aspect was not uniform in this review. By considering the severity of knee OA, it may be possible to examine the efficacy of studies in terms of population and heterogeneity.

Most of the outcomes included in this review were followed up only in the short to medium term. Long-term follow-up, such as up to 12 to 24 months, would help expand our findings with respect to the effectiveness of mHealth.

### Conclusions

Studies on the effectiveness of mHealth in patients with knee OA are increasing. Our review suggests that mHealth is as effective as conventional therapy for pain, physical function, and QoL outcomes. Although the results of this review suggest that mHealth does not have a more significant effect on clinical outcomes than standard rehabilitation or conservative management, this finding is not necessarily negative. mHealth may still be more cost-effective, as it can be as effective as standard care without medical staff supervision or direct face-to-face instruction. In light of the importance of health care affordability, researchers should continue to include cost-effectiveness indicators in future study outcomes.

## Appendix

Multimedia Appendix 1 A comprehensive search strategy for each of the four databases.

Multimedia Appendix 2 A word cloud composed of the included studies.

## Abbreviations

EQ-5D: EuroQol 5 dimensions questionnaire
KOOS-PS: Knee injury and Osteoarthritis Outcome Score-Physical Function Shortform
mHealth: mobile health
OA: osteoarthritis
PICO: patient, intervention, comparison, outcome
PRISMA: Preferred Reporting Items for Systematic Reviews and Meta-Analysis
QoL: quality of life
RCT: randomized controlled trial
SF-36: RAND 36-Item Short Form Health Survey
TUG: Timed Up and Go
WOMAC: Western Ontario and McMaster Universities Osteoarthritis Index

## References

[1] GBD 2019 Diseases and Injuries Collaborators (2020). Global burden of 369 diseases and injuries in 204 countries and territories, 1990-2019: a systematic analysis for the global burden of disease study 2019. Lancet.

[2] Moss RJ, Süle A, Kohl S (2019). eHealth and mHealth. Eur J Hosp Pharm.

[3] Xie S, Wang Q, Wang LQ, Wang L, Song KP, He CQ (2021). Effect of internet-based rehabilitation programs on improvement of pain and physical function in patients with knee osteoarthritis: systematic review and meta-analysis of randomized controlled trials. J Med Internet Res.

[4] Safari R, Jackson J, Sheffield D (2020). Digital self-management interventions for people with osteoarthritis: systematic review with meta-analysis. J Med Internet Res.

[5] Choi W, Zheng H, Franklin P, Tulu B (2019). mHealth technologies for osteoarthritis self-management and treatment: a systematic review. Health Informatics J.

[6] Nelligan RK, Hinman RS, Atkins L, Bennell KL (2019). A short message service intervention to support adherence to home-based strengthening exercise for people with knee osteoarthritis: intervention design applying the behavior change wheel. JMIR mHealth uHealth.

[7] Marcolino MS, Oliveira JAQ, D'Agostino M, Ribeiro AL, Alkmim MBM, Novillo-Ortiz D (2018). The impact of mHealth interventions: systematic review of systematic reviews. JMIR mHealth uHealth.

[8] Cottrell MA, Hill AJ, O'Leary SP, Raymer ME, Russell TG (2017). Service provider perceptions of telerehabilitation as an additional service delivery option within an Australian neurosurgical and orthopaedic physiotherapy screening clinic: a qualitative study. Musculoskelet Sci Pract.

[9] Pelle T, Bevers K, van den Hoogen F, van der Palen J, van den Ende E (2022). Economic evaluation of the Dr. Bart application in individuals with knee and/or hip osteoarthritis. Arthritis Care Res (Hoboken).

[10] Kloek C, Bossen D, Spreeuwenberg PM, Dekker J, de Bakker DH, Veenhof C (2018). Effectiveness of a blended physical therapist intervention in people with hip osteoarthritis, knee osteoarthritis, or both: a cluster-randomized controlled trial. Phys Ther.

[11] Kloek CJJ, van Dongen JM, de Bakker DH, Bossen D, Dekker J, Veenhof C (2018). Cost-effectiveness of a blended physiotherapy intervention compared to usual physiotherapy in patients with hip and/or knee osteoarthritis: a cluster randomized controlled trial. BMC Public Health.

[12] Pelle T, Bevers K, van der Palen J, van den Hoogen FHJ, van den Ende CHM (2020). Effect of the Dr. Bart application on healthcare use and clinical outcomes in people with osteoarthritis of the knee and/or hip in the Netherlands; a randomized controlled trial. Osteoarthritis Cartilage.

[13] Umapathy H, Bennell K, Dickson C, Dobson F, Fransen M, Jones G, Hunter DJ (2015). The web-based osteoarthritis management resource my joint pain improves quality of care: a quasi-experimental study. J Med Internet Res.

[14] Dahlberg LE, Dell'Isola A, Lohmander LS, Nero H (2020). Improving osteoarthritis care by digital means—effects of a digital self-management program after 24- or 48-weeks of treatment. PLoS One.

[15] Kitagawa T, Hirokawa H, Otake N, Denda T, Hiraya K (2021). An mHealth app intervention for self-management of knee osteoarthritis: a protocol for a scoping review. Protocols.io.

[16] Skou ST, Roos EM (2017). Good Life with osteoArthritis in Denmark (GLA:D™): evidence-based education and supervised neuromuscular exercise delivered by certified physiotherapists nationwide. BMC Musculoskelet Disord.

[17] Lespasio MJ, Piuzzi NS, Husni ME, Muschler GF, Guarino A, Mont MA (2017). Knee osteoarthritis: a primer. Perm J.

[18] Ouzzani M, Hammady H, Fedorowicz Z, Elmagarmid A (2016). Rayyan-a web and mobile app for systematic reviews. Syst Rev.

[19] Page MJ, Moher D, Bossuyt PM, Boutron I, Hoffmann TC, Mulrow CD, Shamseer L, Tetzlaff JM, Akl EA, Brennan SE, Chou R, Glanville J, Grimshaw JM, Hróbjartsson A, Lalu MM, Li T, Loder EW, Mayo-Wilson E, McDonald S, McGuinness LA, Stewart LA, Thomas J, Tricco AC, Welch VA, Whiting P, McKenzie JE (2021). PRISMA 2020 explanation and elaboration: updated guidance and exemplars for reporting systematic reviews. BMJ.

[20] Aydogdu O, Uhud S, Zübeyir S (2019). AB1418-HPR mobile-phone-based home exercise training program in patients with knee osteoarthritis. Ann Rheum Dis.

[21] Hsu YI, Chen YC, Lee CL, Chang NJ (2021). Effects of diet control and telemedicine-based resistance exercise intervention on patients with obesity and knee osteoarthritis: a randomized control trial. Int J Environ Res Public Health.

[22] Hussain MS, Li J, Brindal E, van Kasteren Y, Varnfield M, Reeson A, Berkovsky S, Freyne J (2017). Supporting the delivery of total knee replacements care for both patients and their clinicians with a mobile app and web-based tool: randomized controlled trial protocol. JMIR Res Protoc.

[23] Pronk Y, Peters MCWM, Sheombar A, Brinkman J (2020). Effectiveness of a mobile ehealth app in guiding patients in pain control and opiate use after total knee replacement: randomized controlled trial. JMIR mHealth uHealth.

[24] Rafiq MT, Abdul Hamid MS, Hafiz E (2021). The effect of rehabilitation protocol using mobile health in overweight and obese patients with knee osteoarthritis: a clinical trial. Adv Rheumatol.

[25] Seward MW, Antonelli BJ, Giunta N, Iorio R, Fitz W, Lange JK, Shah VM, Chen AF (2020). Weight loss before total joint arthroplasty using a remote dietitian and mobile app: study protocol for a multicenter randomized, controlled trial. J Orthop Surg Res.

[26] Stauber A, Schüßler N, Palmdorf S, Schürholz N, Bruns D, Osterbrink J, Nestler N (2020). RECOVER-E - a mobile app for patients undergoing total knee or hip replacement: study protocol. BMC Musculoskelet Disord.

[27] Wang L, Xie S, Bao T, Zhu S, Liang Q, Wang X, Zhang R, Xiang X, Du C, He C (2021). Exercise and education for community-dwelling older participants with knee osteoarthritis: a video-linked programme protocol based on a randomised controlled trial. BMC Musculoskelet Disord.

[28] Nelligan RK, Hinman RS, Kasza J, Crofts SJC, Bennell KL (2021). Effects of a self-directed web-based strengthening exercise and physical activity program supported by automated text messages for people with knee osteoarthritis: a randomized clinical trial. JAMA Intern Med.

[29] Skrepnik N, Spitzer A, Altman R, Hoekstra J, Stewart J, Toselli R (2017). Assessing the impact of a novel smartphone application compared with standard follow-up on mobility of patients with knee osteoarthritis following treatment with Hylan G-F 20: a randomized controlled trial. JMIR Mhealth Uhealth.

[30] Pelle T, van der Palen J, de Graaf F, van den Hoogen FHJ, Bevers K, van den Ende CHM (2021). Use and usability of the Dr. Bart app and its relation with health care utilisation and clinical outcomes in people with knee and/or hip osteoarthritis. BMC Health Serv Res.

[31] Xie SH, Wang Q, Wang LQ, Zhu SY, Li Y, He CQ (2020). The feasibility and effectiveness of internet-based rehabilitation for patients with knee osteoarthritis: a study protocol of randomized controlled trial in the community setting. Medicine (Baltimore).

[32] Harmelink KEM, Zeegers AVCM, Tönis TM, Hullegie W, Nijhuis-van der Sanden MWG, Staal JB (2017). The effectiveness of the use of a digital activity coaching system in addition to a two-week home-based exercise program in patients after total knee arthroplasty: study protocol for a randomized controlled trial. BMC Musculoskelet Disord.

[33] Pelle T, Bevers K, van der Palen J, van den Hoogen FHJ, van den Ende CHM (2019). Development and evaluation of a tailored e-self-management intervention (Dr. Bart app) for knee and/or hip osteoarthritis: study protocol. BMC Musculoskelet Disord.

