# Supplementary material for: mHealth for the Self-management of Knee Osteoarthritis: Scoping Review
Source: J Med Internet Res. 2023 May 8;25:e38798. doi: 10.2196/38798 (PMC10203920; doi:10.2196/38798)
Supplement: Multimedia Appendix 1 [file jmir_v25i1e38798_app1.docx]

## Appendix

A comprehensive search strategy for each of the four databases

#### PubMed search strategy

#### Knee osteoarthritis keywords: (knee osteoarthritis [MeSH] OR (arthrosis OR arthritis OR osteoarthr* OR OA OR degeneration OR degenerative OR arthralgia) AND knee)

#### mHealth keywords: ((((smart OR mobile) and phone) AND (app OR application)) OR (eHealth OR e-health) OR (mHealth OR m-health) OR digital health OR (personal digital assistant*) OR (tablet* computer*) OR (ipad* OR iphone* OR ipod*) OR (ios OR android) OR wearable OR sensor)

#### Self-management keywords: ((strength* OR isometric* OR isotonic* OR isokinetic* OR aerobic* OR endurance OR weight*) OR (exercise OR training) OR sport OR (muscle strength*) OR (physical endurance) OR rehabilitation OR (selfmanagement OR self-management) OR (Self Care OR self-care) OR pain OR function OR (quality of life OR QOL) OR (control OR management) OR treatment OR (life OR lifestyle) OR support)

#### Web of Science search strategy

#### Knee osteoarthritis keywords: ((osteoarthritis OR arthrosis OR arthritis OR OA OR degeneration OR degenerative OR arthralgia) AND knee)

#### mHealth keywords: (((smart OR mobile) and phone) AND (app OR application)) OR (eHealth OR e-health) OR (mHealth OR m-health) OR digital health OR (tablet* computer*) OR (ipad* OR iphone* OR ipod*) OR (ios OR android) OR wearable OR sensor

#### Self-management keywords: ((strength* OR aerobic* OR endurance OR weight*) OR (exercise OR training) OR sport OR (muscle strength*) OR rehabilitation OR self-management OR self-care OR pain OR function OR QOL OR (control OR management) OR treatment)

#### Cumulative Index to Nursing and Allied Health Literature (CINAHL) search strategy

#### Knee osteoarthritis keywords: knee osteoarthritis OR ((arthrosis OR arthritis OR osteoarthritis OR OA OR degeneration OR degenerative OR arthralgia)) AND knee

#### mHealth keywords: ((((smart OR mobile) and phone) AND (app OR application)) OR (eHealth OR e-health) OR (mHealth OR m-health) OR digital health OR (personal digital assistant) OR (tablet computer) OR (ipad OR iphone OR ipod) OR (ios OR android) OR wearable OR sensor)

#### Self-management keywords: ((strength OR isometric OR isotonic OR isokinetic OR aerobic OR endurance OR weight) OR (exercise OR training) OR sport OR (muscle strength) OR (physical endurance) OR rehabilitation OR (selfmanagement OR self-management) OR (self care OR self-care) OR pain OR function OR (quality of life OR QOL) OR (control OR management) OR treatment OR (life OR lifestyle) OR support)

#### Cochrane Central Register of Controlled Trials (CENTRAL) search strategy

Knee osteoarthritis keywords: knee osteoarthritis OR ((arthrosis OR arthritis OR osteoarthritis OR OA OR degeneration OR degenerative OR arthralgia)) AND knee

mHealth keywords: ((((smart OR mobile) and phone) AND (app OR application)) OR (eHealth OR e-health) OR (mHealth OR m-health) OR digital health OR (personal digital assistant) OR (tablet computer) OR (ipad OR iphone OR ipod) OR (ios OR android) OR wearable OR sensor)

Self-management keywords: ((strength OR isometric OR isotonic OR isokinetic OR aerobic OR endurance OR weight) OR (exercise OR training) OR sport OR (muscle strength) OR (physical endurance) OR rehabilitation OR (selfmanagement OR self-management) OR (self care OR self-care) OR pain OR function OR (quality of life OR QOL) OR (control OR management) OR treatment OR (life OR lifestyle) OR support)
