# Supplementary figures and images for: mHealth for the Self-management of Knee Osteoarthritis: Scoping Review
Source: J Med Internet Res. 2023 May 8;25:e38798. doi: 10.2196/38798 (PMC10203920; doi:10.2196/38798)

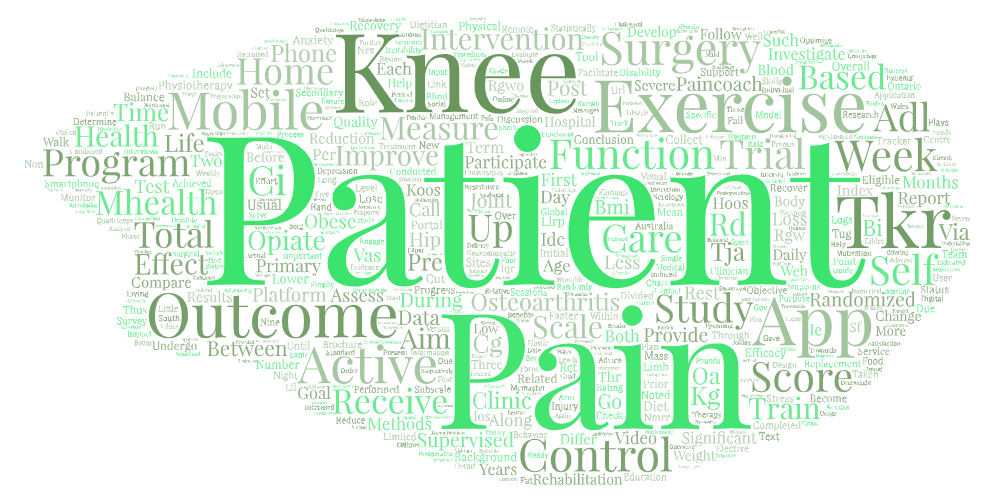

Supplement: Multimedia Appendix 2 [file jmir_v25i1e38798_app2.png]
